# Patient Retention in Antiretroviral Therapy Programs in Sub-Saharan Africa: A Systematic Review

**DOI:** 10.1371/journal.pmed.0040298

**Published:** 2007-10-16

**Authors:** Sydney Rosen, Matthew P Fox, Christopher J Gill

**Affiliations:** 1 Center for International Health and Development, Boston University School of Public Health, Boston, Massachusetts, United States of America; 2 Health Economics Research Office, Wits Health Consortium, Johannesburg, South Africa; 3 Infectious Diseases Section, Department of Medicine, Boston Medical Center, Boston, Massachusetts, United States of America; Mexican National Institutes of Health, Mexico

## Abstract

**Background:**

Long-term retention of patients in Africa's rapidly expanding antiretroviral therapy (ART) programs for HIV/AIDS is essential for these programs' success but has received relatively little attention. In this paper we present a systematic review of patient retention in ART programs in sub-Saharan Africa.

**Methods and Findings:**

We searched Medline, other literature databases, conference abstracts, publications archives, and the “gray literature” (project reports available online) between 2000 and 2007 for reports on the proportion of adult patients retained (i.e., remaining in care and on ART) after 6 mo or longer in sub-Saharan African, non-research ART programs, with and without donor support. Estimated retention rates at 6, 12, and 24 mo were calculated and plotted for each program. Retention was also estimated using Kaplan-Meier curves. In sensitivity analyses we considered best-case, worst-case, and midpoint scenarios for retention at 2 y; the best-case scenario assumed no further attrition beyond that reported, while the worst-case scenario assumed that attrition would continue in a linear fashion. We reviewed 32 publications reporting on 33 patient cohorts (74,192 patients, 13 countries). For all studies, the weighted average follow-up period reported was 9.9 mo, after which 77.5% of patients were retained. Loss to follow-up and death accounted for 56% and 40% of attrition, respectively. Weighted mean retention rates as reported were 79.1%, 75.0% and 61.6 % at 6, 12, and 24 mo, respectively. Of those reporting 24 mo of follow-up, the best program retained 85% of patients and the worst retained 46%. Attrition was higher in studies with shorter reporting periods, leading to monthly weighted mean attrition rates of 3.3%/mo, 1.9%/mo, and 1.6%/month for studies reporting to 6, 12, and 24 months, respectively, and suggesting that overall patient retention may be overestimated in the published reports. In sensitivity analyses, estimated retention rates ranged from 24% in the worse case to 77% in the best case at the end of 2 y, with a plausible midpoint scenario of 50%.

**Conclusions:**

Since the inception of large-scale ART access early in this decade, ART programs in Africa have retained about 60% of their patients at the end of 2 y. Loss to follow-up is the major cause of attrition, followed by death. Better patient tracing procedures, better understanding of loss to follow-up, and earlier initiation of ART to reduce mortality are needed if retention is to be improved. Retention varies widely across programs, and programs that have achieved higher retention rates can serve as models for future improvements.

## Abstract 2

Almost half of people entering African HIV treatment programs were lost to follow-up or died within two years, according to this systematic review by Sydney Rosen and colleagues.

## Editors' Summary

**Background.:**

About 25 million people in sub-Saharan Africa are infected with the human immunodeficiency virus (HIV), the cause of acquired immunodeficiency syndrome (AIDS). Every year, about three million more people become infected with HIV and 2 million die from AIDS in this region, where the pandemic has reduced life expectancy, orphaned many children, and reversed economic growth. Since 1996, HIV-positive people living in wealthier parts of the world have had access to cocktails of antiretroviral drugs that hold HIV in check and allow them to live relatively normal, healthy lives. But these drugs are expensive and it is only in the past five years that antiretroviral therapy (ART) programs have been initiated in sub-Saharan Africa, often with international support.

**Why Was This Study Done?:**

For ART to work, HIV-infected individuals whose immune systems have been damaged by the virus have to take antiretroviral drugs regularly for the rest of their lives. If people take ART irregularly or stop taking their medications they may become sicker or die, or the viruses they carry may become resistant to antiretroviral drugs. Several studies have looked at how well patients on ART stick to their day-to-day medication schedules, but how long patients stay in treatment programs, which they must do to prevent illness and death from AIDS, has received little attention. In this study the researchers reviewed reports of whether patients stay in treatment in ART programs in sub-Saharan Africa, and also looked at the reasons why they drop out.

**What Did the Researchers Do and Find?:**

The researchers identified 32 scientific reports published or presented at meetings between 2000 and 2007 that gave details of the proportion of adult patients retained (alive and receiving ART) in ART treatment programs (not including research studies) in 13 countries in sub-Saharan Africa. The average follow-up time of the programs (adjusted for number of patients in each) was 9.9 months. At this time, 77.5% of the patients were retained on average. Of the patients not retained, just under half had died and half had been lost to follow up. That is, they had missed clinic visits or had not picked up their medication. Estimated average retention rates at 6, 12, and 24 months were 79.08%, 75% and 61.6%, respectively; retention rates reported at 24 months ranged between 46% and 85% of patients. Finally, using sensitivity analysis (a technique that can estimate best- and worst-case possibilities), the researchers estimated that actual retention in ART programs after 2 years probably lies between one-quarter and three-quarters of patients.

**What Do These Findings Mean?:**

These results show that roughly half of people starting HIV treatment programs in Africa are no longer receiving treatment after two years. The overall success rates of African treatment programs may actually be even lower, if one takes into account that programs with very low retention may be unlikely to publish their results. This study therefore indicates that a worrying number of patients in sub-Saharan Africa who need ART are lost from treatment programs. Because many of these patients are lost because they die from AIDS, one way to improve retention might be to start treating people with ART earlier, before they become seriously ill from HIV. Better efforts to find out exactly why patients drop out of programs (for example, the cost of drugs and/or of transport to clinics) might reduce the number of patients lost to follow up. The researchers also suggest that ART programs with very high retention rates might serve as models to improve retention rates in other programs.

**Additional Information.:**

Please access these Web sites via the online version of this summary at http://dx.doi.org/10.1371/journal.pmed.0040298.

- Information is available from the US National Institute of Allergy and Infectious Diseases on HIV infection and AIDS
- HIV InSite is a regional page on sub-Saharan Africa from the University of California, San Francisco
- Information is provided by the US Centers for Disease Control and Prevention on the US President's Emergency Plan for AIDS Relief in various countries and regions
- Avert is an international AIDS charity that provides information on HIV and AIDS in Africa
- Aidsmap is an international AIDS organization that summarizes research about HIV/AIDS and reports news (in English, Spanish, Portuguese, French, and Russian)

## Introduction

In the half decade since the first large-scale antiretroviral treatment (ART) programs for HIV/AIDS were launched in sub-Saharan Africa, much attention has focused on patients' day-to-day adherence to antiretroviral (ARV) medications [1–3]. Long-term retention of patients in treatment programs, a prerequisite for achieving any adherence at all, has received far less attention. Perhaps because most large scale treatment providers have few resources available to track missing patients, most studies treat patient attrition as a side issue and focus solely on describing those patients who are retained. Moreover, adherence can be assessed over very short periods, whereas long-term retention requires, by definition, long-standing programs.

Attrition from antiretroviral treatment programs is generally divided into four categories. The two most common are (1) the death of the patient—several studies have reported high rates of early mortality—and (2) “loss to follow-up,” a catch-all category for patients who miss scheduled clinic visits or medication pickups for a specified period of time. Some patients remain in care but stop taking ARV medications (3). Others transfer to other facilities and continue on ART (4).

Treatment discontinuation raises some of the same concerns about drug resistance that incomplete adherence does and, even worse, negates much of the benefit sought by those implementing treatment programs. Patients with clinical AIDS who discontinue ART will likely die within a relatively short time [4]. High rates of attrition from treatment programs thus pose a serious challenge to program implementers and constitute an inefficient use of scarce treatment resources. In this study, we analyzed reported treatment program retention and attrition in sub-Saharan Africa in order to document the magnitude of the problem and help policy makers and program managers address the challenge of patient retention.

## Methods

### Definitions

For this review, “retention” refers to patients known to be alive and receiving highly active ART at the end of a follow-up period. “Attrition” is defined as discontinuation of ART for any reason, including death, loss to follow-up, and stopping ARV medications while remaining in care. Transfer to another ART facility, where reported, is not regarded as attrition—patients who transfer are assumed to be retained. We accepted the varying definitions of loss to follow-up used by the respective studies. Many studies considered patients lost if they were more than 3 mo late for a scheduled consultation or medication pickup, but some studies used more or less stringent definitions ranging from 1 to 6 mo late for a scheduled consultation or medication pick-up.

### Inclusion and Exclusion Criteria

Studies were included in the review if they reported the proportion of adult HIV-1 patients retained in highly active ART programs implemented in service delivery (nonresearch) settings in sub-Saharan Africa. All patients who initiated ART had to be included in the report, not just those still in care at the time of censoring (i.e., only intention-to-treat analyses were included). Clinical trials, including Phase 3 trials, were excluded, although some subjects of reviewed studies transferred into the treatment program from a clinical trial. A median follow-up period of at least six full months (26 weeks) was also required. Studies that reported mortality but not other categories of attrition and studies that reported only on-treatment analyses, or where we were unable to determine whether the study was intention-to-treat or not, were also excluded. A few of the reviewed studies did not differentiate between adult and pediatric patients; those that considered only pediatric patients were excluded.

### Search Strategy

To identify eligible studies, we conducted a systematic search of the English-language published literature, gray literature (project reports available online), and conference abstracts between 2000 and 2007. The search included Ovid Medline (1996 to July 2007), EMBASE (inception to July 2007), ISI Web of Science (August 2002 to July 2007), the Cumulative Index to Nursing & Allied Health Literature (2002 to July 2007), and the Cochrane Database of Systematic Reviews (inception to second quarter 2007). We also searched the abstracts of the conferences of the International AIDS Society (inception to 2006), the Conference on Retroviruses and Opportunistic Infections (inception to 2007), the HIV Implementers' Meetings (2006–2007), and the South African AIDS Conference (2005–2007). The bibliographies of five recently published reviews of treatment outcomes, mortality, or ARV adherence in resource-constrained settings were also searched [1–3,5,6]. Our search strategy combined the terms “antiretroviral” and “Africa” or “developing countries” with each of retention/attrition/loss to follow-up/mortality/evaluation/efficacy. When more than one source reported on the same cohort of patients, the source containing the most detailed data about retention and attrition or the longest follow-up period was selected for the review. Although non-English databases were not searched, English-language abstracts of non-English papers identified in our search were included. Eligible studies were identified by the first author (SR) and eligibility confirmed by the other authors (MF and CG).

It should be noted that the Antiretroviral Therapy in Low Income Countries (ART-LINC) collaboration has recently reported aggregate 1 y mortality and loss to follow-up rates for 13 cohorts in sub-Saharan Africa [5]. Some of the patients in these cohorts are included in the studies reviewed here. To avoid duplication, findings from the ART-LINC cohorts were not included in this analysis but are noted in the discussion.

### Data Analysis

Most studies reported patient attrition at months 6, 12, and/or 24 after treatment initiation. We therefore used these same intervals in this analysis. For papers that reported on intervals other than 6, 12, or 24 mo, we classified the reported attrition rate using the nearest time point. If the report did not list attrition rates by time, but did list a median duration of observation, we estimated attrition at the 6, 12, or 24 mo interval closest to the reported observation period. In some cases, follow-up periods and/or retention rates were calculated by the authors using data provided in the article or extracted from figures (e.g. Kaplan-Meier survival curves). Where appropriate, we calculated weighted averages for demographic features of the cohort participants or other factors related to the studies. For proportions, averages were weighted by the inverse of their variances [1 ÷ (*p* × [1 − *p*] ÷ *n*), where *p* is the proportion and *n* is the sample size]. Because we did not have the individual patient data for continuous variables nor their standard deviations, we were unable to calculate variances for these variables. In these situations, we weighted by cohort size.

In some instances, studies reported follow-up to 12 or 24 mo but did not report on intermediate retention rates. In plotting attrition for such studies over time we used extrapolated values, taking the midpoint between the known adjacent values. For example, if a study reported to 12 mo but did not report the 6 mo value, we defined the 6 mo value as the midpoint between 0 and 12 mo, with 100% at baseline representing all of those who initially started therapy. We calculated weighted average attrition rates at each interval (6, 12, and 24 mo) for the reported numbers of participants remaining when using reported values and for the estimated numbers of participants remaining when using extrapolated values. Selected demographic variables relating cohort or program characteristics to attrition rates were analyzed using linear regression. Because only a few studies reported beyond 24 mo, we were unable to calculate any meaningful summary statistics beyond the 24 mo mark.

To estimate aggregate average attrition rates at 6, 12, and 24 mo we used several approaches. Attrition for each program was plotted separately and attrition rates calculated as the percentage of patients lost per month. We also plotted Kaplan-Meier survival curves using the 6, 12, and 24 mo intervals as the step-down points. Fewer studies presented 12 mo data than 6 mo data, however, and fewer still contained 24 mo data. Many of the studies with the highest attrition contributed data only for the shorter time intervals. Given the concern that shorter durations of reporting could be associated with lower rates of patient retention, we also conducted sensitivity analyses to model possible future retention. For the best-case scenario, we optimistically assumed that no further attrition would occur beyond the last reported observation and extrapolated the last reported retention value forward to 24 mo. In the worst-case scenario, we extrapolated the slope of attrition forward in time, assuming that each cohort's attrition would continue along the same slope from the last reported observation to month 24. We assigned a lower limit of 0% in those situations where the estimated future retention rate fell below 0%. Our midpoint scenario was the mean of the best- and worst-case scenarios. Analyses were conducted using Excel, SAS version 8.2, and SPSS version 11.0.

## Results

**Figure 1:**
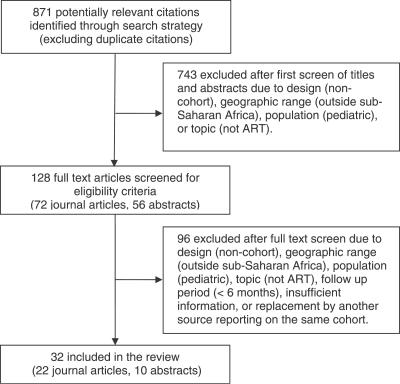
Study Flow Chart

We included 32 publications reporting on 33 patient cohorts totaling 74,289 patients in 13 countries in our analysis. These studies were selected from a total of 871 potentially relevant, unique citations identified in our search (Figure 1).

Table 1 summarizes key features of the studies, including the sites at which they were conducted. Not all of the publications reported all the details we sought about program and patient characteristics and retention, but all provided at least one indicator of patient retention after a median follow-up period of at least 6 mo. The studies report on patients who initiated ART as early as 1996, though most enrolled their cohorts between 2001 and 2004. The studies were published or presented between 2002 and 2007, with the majority appearing as peer reviewed articles in 2006 or 2007. Most of the programs were implemented by the public sector (17 of 33, 52%). Of 33 cohorts, 15 (45%) fully subsidized the cost of ART; six (18%) were partially subsidized; and six (18%) required patients to pay fully for their care; the rest did not report their payment structure. Roughly half were single-site programs (15 of 33, 45%); multi-site programs contributed data from between two and 69 sites.

**Table 1:**
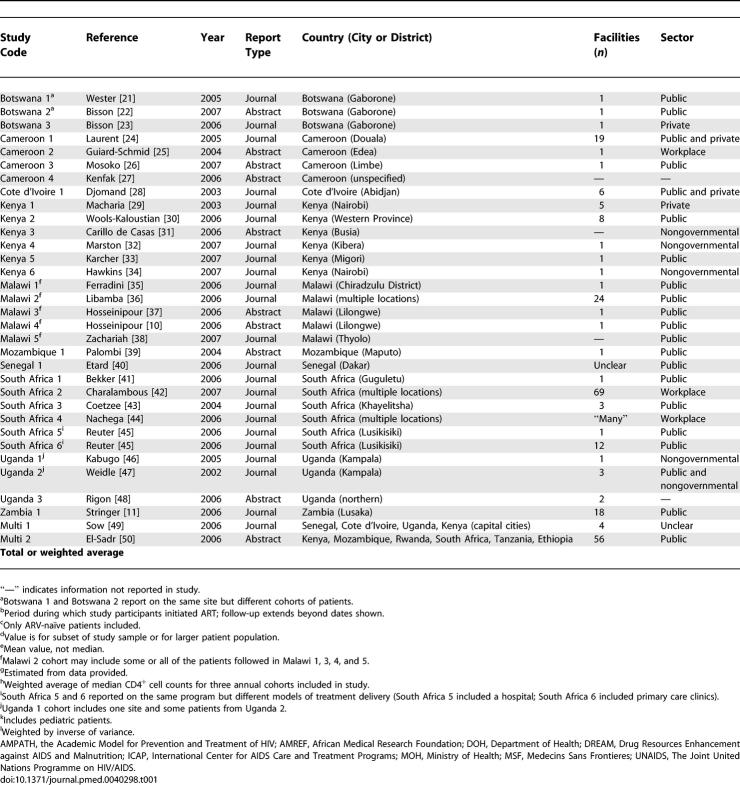
Characteristics of Antiretroviral Treatment Programs and Patient Cohorts Included in This Analysis (extended on next page)

**Table 1:**
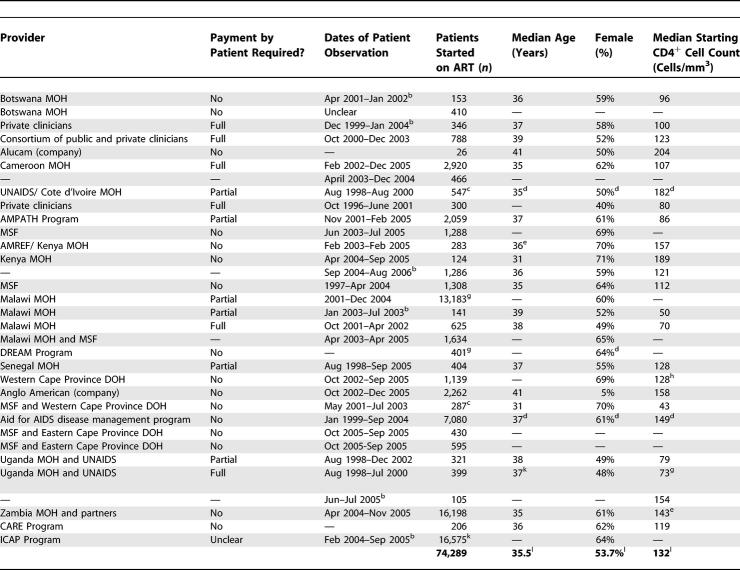
Extended.

Table 1 also provides the population characteristics of the cohorts studied. The weighted mean age of the cohort participants was 35.5 y, and 53.7% of all patients were female (range 6%–70%). All but one cohort had median starting CD4^+^ T cell counts at or well below 200 × 10^3^ cells/mm^3^, with a weighted mean starting CD4^+^ T cell count of 132 × 10^3^ cells/mm^3^ (range 43–204).

Table 2 presents the proportion of patients from each cohort who remained alive and under treatment with antiretroviral medications, transferred to another treatment facility, died, were lost to follow-up, or discontinued treatment with ARVs but remained in care at the end of the median follow-up period. Bearing in mind that we excluded studies with less than 6 mo median follow-up, the weighted average follow-up was 9.9 mo, after which time overall retention of patients alive, in care, and on ART was 77.5%.

**Table 2:**
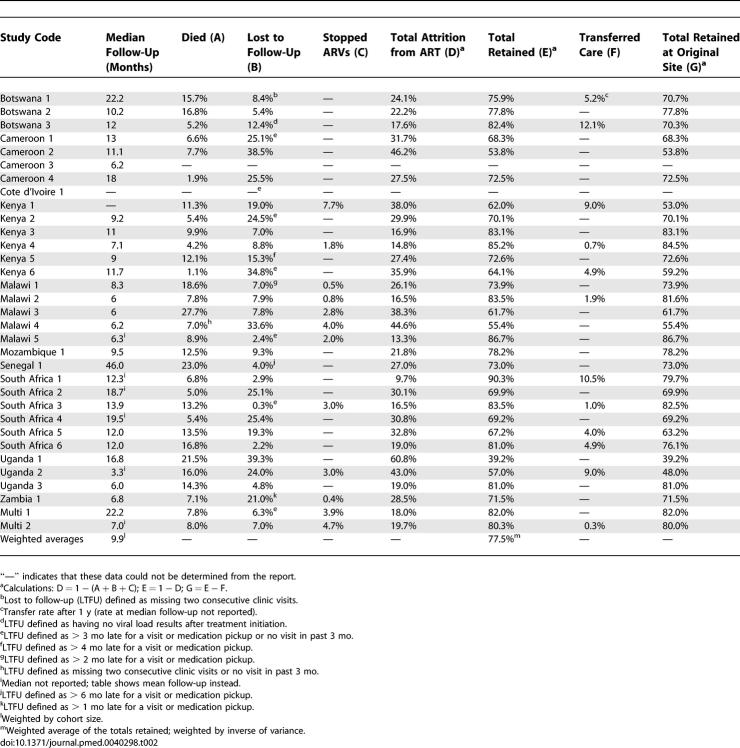
Median Follow-Up and Rates of Patient Attrition, as Reported, from Antiretroviral Treatment Programs

Across all the cohorts, the largest contributor to attrition was loss to follow-up (56% of attrition), followed by death (40% of attrition). The widely varying definitions of loss to follow-up used by the studies are indicated in Table 2. A small fraction (4% of attrition) discontinued ART but remained under care at the same site.

Table 3 reports overall retention at 6, 12, and 24 mo. SA 1 had the highest retention at 12 mo. While this program did not report for 6 mo, at 12 mo its retention of 90% was still higher than the highest reported value among the programs that reported their 6 mo outcomes. The programs with the lowest retention at each time point were Malawi 4 (55%) at 6 mo; Uganda 2 (49%) at 12 mo; and Uganda 1 (46%) at 24 mo. Malawi 4 did not report beyond 6 mo and Uganda 2 did not report beyond 12 mo, but were both on a trajectory toward even lower retention rates at the later time points.

**Table 3:**
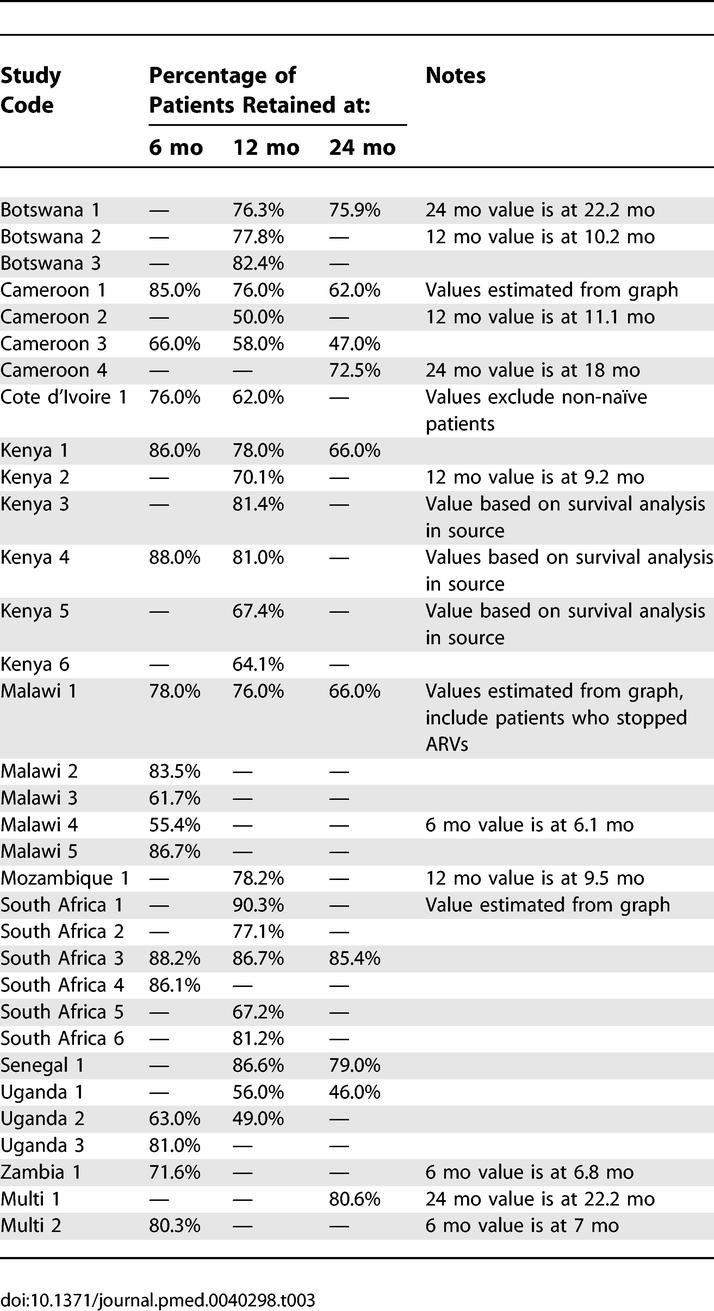
Retention of Patients at 6, 12, and 24 Months after Initiation of ART

Using linear regression, we found no association between 6 mo attrition rates and cohort size (*p* = 0.32), attrition and baseline CD4^+^ cell counts (*p* = 0.72), proportion of women (*p* = 0.23), or year of program initiation (*p* = 0.40). Programs that required no payment had higher retention rates at 6 mo compared to those requiring partial or full payment (86.5% versus 76.7%, *p* = 0.01).

**Figure 2:**
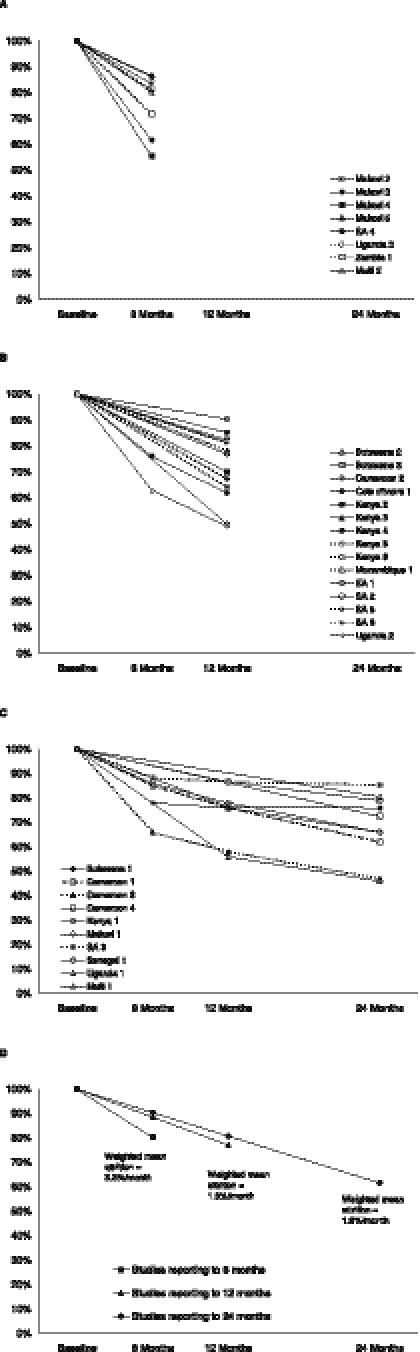
Attrition Rates by Reporting Duration (A) Studies reporting to 6 mo median follow-up. (B) Studies reporting to 12 mo median follow-up. (C) Studies reporting to 24 mo median follow-up. (D) Weighted mean attrition rates by duration cohort. SA, South Africa.

Figures 2A–2C plot attrition rates for each cohort separately. The studies are clustered on the basis of duration of reporting. By 6 mo, 9 of 33 cohorts (27%) had 20% or greater attrition rates; by 12 mo this proportion had risen to 16 of 25 reporting cohorts (64%).

The weighted mean retention rates as reported in the studies were 79.8% at 6 mo, 75.1% at 12 mo, and 61.6% at 24 mo. As an alternative approach, we also plotted Kaplan-Meier survival curves at months 6, 12, and 24 for all the studies combined. The largest fall-off occurred between 6 and 12 mo; overall retention was approximately 89% by 6 mo, 70% by 1 year, and just under 60% by 2 years.

Four of the eight studies in Figure 2A with attrition of at least 20% at 6 mo included data only to 6 mo. Similarly, of the cohorts with data at 12 mo and attrition of 25% or more, six of 11 did not extend beyond 12 mo. We therefore calculated the slopes for attrition rates for each group of cohorts in Figure 2A–2C separately, to determine if the average monthly attrition rates differed as the duration of reporting increased. As shown in Figure 2D, the weighted mean attrition rates were 3.3%/month, 1.9%/month, and 1.6%/month for studies reporting to 6 mo, 12 mo, and 24 mo, respectively, raising the possibility that shorter durations of reporting were associated with lower retention rates.

**Figure 3:**
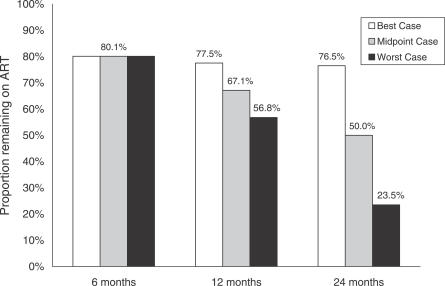
Sensitivity Analysis for Attrition

Given this apparent reporting bias, we were concerned that reporting average retention rates using the simple aggregate weighted averages reported above would overestimate actual retention. We therefore conducted sensitivity analyses to model attrition rates under three different scenarios. As shown in Figure 3, all three scenarios are the same at 6 mo, with approximately 80% retention. Under the best-case scenario, further attrition would be negligible, with more than 76% still retained by the end of 2 y. Under the worst-case scenario, 76% of patients would be lost by 2 y. The midpoint scenario predicted patient retention of 50% by 2 y.

## Discussion

The analysis presented here suggests that ART programs in Africa are retaining, on average, roughly 80% of their patients after 6 mo on ART and between one-fourth and three-fourths of their patients by the end of 2 y, depending on the estimating method used. Prior to the availability of ART in Africa, the median interval from HIV infection to AIDS-related death was under 10 y; once a patient was diagnosed with AIDS, median survival was less than 1 y [7]. Since most patients in Africa initiate ART only after an AIDS diagnosis, most ART patients would have died within a year had antiretroviral therapy not been available. Each patient who is retained in care and on ART can thus be regarded as a life saved and a source of tremendous benefit to patients' families and communities.

For those who have struggled to launch and expand treatment programs in resource-constrained settings, reaching a 60% patient retention—and thus survival—rate after two years of treatment, as estimated by the Kaplan-Meier survival analysis, in just a few years' time is an extraordinary accomplishment. It is also noteworthy in the global context: in developed countries, adherence to medication for chronic diseases in general averages only 50% [8]. Similarly, treatment completion rates for tuberculosis, which requires a temporary rather than permanent commitment to adherence and a less demanding dosing schedule, average 74% in the African region, with a range among countries from 22% to 94% [9]. Taken in the context of medication adherence in general, the record of African ART programs lies within the bounds of previous experience.

At the same time, however, losing up to half of those who initiate ART within two years is cause for concern. From the data as reported, attrition averaged roughly 22% at 10 mo of follow-up. This average comprised mainly deaths (40% of attrition) and losses to follow-up (56%). In comparison, the ART-LINC Collaboration, which analyzed data from 18 cohorts across the developing world, reported loss to follow-up rates among the 13 sub-Saharan African cohorts averaging 15% (range 0%–44%) in the first year after initiation; mortality averaged 4.2% across all 18 cohorts (African regional rate not provided) [5].

On the basis of our survival and sensitivity analyses, we believe that actual attrition is higher than the 22% average we report, mainly because the programs with the highest attrition were least likely to provide data beyond the first 6 mo of ART. There are several plausible explanations for the higher attrition seen among programs with shorter durations of reporting. One possibility is that limited availability of resources to a given program could affect both its ability to retain patients and to conduct long-term surveillance of its outcomes. Another, less pessimistic explanation is that shorter durations of reporting reflect newer programs that are still in the process of developing optimal strategies for patient retention: had they reported at a later point in their implementation, retention rates might have been higher. The magnitude of the under-reporting bias is also uncertain, although our sensitivity analysis gives a plausible range between two implausible extremes (the best case being implausible because it assumes zero further attrition beyond the point of last reporting, and the worst case because it assumes that there will be constant attrition over time, rather than reaching a plateau or at least slowing substantially). The midpoint scenario suggests that approximately half of all patients started on ART were no longer on treatment at the end of two years.

One of the principal challenges to this analysis is interpreting the large proportion of attrition from “loss to follow-up.” Some of these patients undoubtedly represent unrecorded deaths, but others may be patients who identified alternative sources for ART or had taken an extended “break” from therapy, to which they will return when their condition worsens again or they obtain the financial resources needed for transport or clinic fees. One study in Malawi discovered, for example, that 24% of patients originally recorded as lost to follow-up re-enrolled at the same site two years later when ART became free of charge [10]. For some of the studies included in this analysis, on the other hand, the unrecorded death explanation is more persuasive. For example, the Zambia 1 cohort of more than 16,000 patients reported 21% loss to follow-up after approximately 6 mo [11]. The scope and scale of this program means that it is the primary source of ART in Lusaka, making it unlikely that most of the estimated 3,300 lost patients could have found alternative sources of care. A recent attempt to trace lost-to-follow-up patients in Malawi determined that 50% had died, 27% could not be found, and most of the rest had stopped ART [12].

Because those reporting on these cohorts do not know what ultimately happened to patients categorized as lost to follow-up, high loss to follow-up rates can have varied interpretations. A good deal of research on barriers to adherence and reasons for treatment discontinuation has been published [13]. Important barriers to adherence include cost of drugs and/or transport, fear of disclosure or stigma, and side effects [14,15]. Some of these barriers can be addressed relatively easily, for example by providing transport vouchers to ensure that patients can attend the clinic; others, such as stigma, require more profound changes. In any case, high reported rates of loss to follow-up are a strong call to improve patient tracing procedures, to minimize the number of patients who fall into the difficult-to-address category of “lost, reason unknown.”

Given that the long-term prognosis of ART patients is inversely related to starting CD4^+^ T cell counts [16,17], an additional issue to consider is the low median starting CD4^+^ cell counts reported by every one of the studies in this analysis. This problem has been identified previously, particularly in South Africa [18–20]. The analysis here makes clear that the problem is nearly universal in Africa and cuts across all types of treatment programs. It is evident in the high death rates reported by some studies after only a few months of follow-up, such as Malawi 3.

There is a high degree of heterogeneity in retention rates between the different cohorts in our analysis and among categories of attrition. Some programs appear to have been highly successful in retaining patients, while others clearly struggled to do so. Some programs have suffered high mortality rates but low loss to follow-up, others the opposite. Early mortality, which may be largely due to the late stage at which many patients present for treatment, requires interventions different from those needed to address later loss to follow-up, about which very little is known. Interventions to address the various types of attrition must thus be tailored to local circumstances. The success of some programs with very high retention may provide examples that others can follow. The findings here can thus be seen as a part of an ongoing process to identify and solve problems within existing treatment programs, even as we expand their scope and launch new ones.

Our analysis has a number of limitations, chiefly that incomplete reporting forced us to extrapolate some values. Extrapolating backward assumes that attrition rates are distributed linearly over time, which is unlikely to be the case. Evidence from this and other studies suggest that the highest attrition occurs during the first 6 mo. However, this limitation only pertains to the shape of the attrition curves, not to their final end points. Extrapolating forward, which we used only in the sensitivity analysis to establish the hypothetical “worst case” scenario, also suffers from this limitation, compounded by the fact that our confidence in the forward boundary is limited.

In addition, our analysis is necessarily limited to publicly available reports and thus potentially subject to publication bias. Researchers may be less inclined to publish long-term outcomes from cohorts that have experienced very high early attrition. It is also likely that programs with better access to resources, both financial and human, are also better able to monitor, analyze, and publish their results. Our aggregate findings may thus represent the better-resourced programs in Africa.

In conclusion, African ART programs are retaining about 60% of their patients in the first two years. This average masks a great deal of heterogeneity, however. At one end of the spectrum represented by the reviewed studies, two-year retention neared 90%; at the other end, attrition reached 50%. Better information on those who are lost to follow-up is urgently needed. Since losses to follow-up account for the majority of all attrition in more than half of the studies reviewed, the problem of attrition cannot be addressed effectively without better means to track patients. Only then can we address the pressing question of why patients drop out and what conditions, assistance, or incentives will be needed to retain them.

## Supporting Information

Protocol S1 Search Protocol (85 KB DOC)

## Abbreviations

ART: antiretroviral therapy
ARV: antiretroviral
