# Supplementary material for: Patient Retention in Antiretroviral Therapy Programs in Sub-Saharan Africa: A Systematic Review
Source: PLoS Med. 2007 Oct 16;4(10):e298. doi: 10.1371/journal.pmed.0040298 (PMC2020494; doi:10.1371/journal.pmed.0040298)
Supplement: Supplementary file 1 [file pmed.0040298.sd001.doc]

**Search protocol for systematic review of patient retention in antiretroviral therapy programs in sub-Saharan Africa**

Authors

Sydney Rosen (lead)

Matthew Fox

Christopher Gill

Background/justification

Although a good deal of research on day-to-day adherence to antiretroviral therapy in sub-Saharan Africa has been published, long-term retention of patients in treatment programs has received relatively little attention. Some published and gray reports of program outcomes include data on patient retention in the first four or five years of treatment, however. Since long-term retention of patients is essential for success, a review of experience to date would be useful.

Review questions

1. What is the rate of all-cause adult patient attrition from antiretroviral therapy programs implemented in service delivery settings in sub-Saharan Africa?
2. What proportion of adult patient attrition can be attributed to mortality, stopping of ARVs, and loss to follow up?
3. Do attrition rates change as the duration on ART increases?

Search strategy

*Electronic databases*

| Database | Search terms |
| --- | --- |
| Medline (Ovid) | - antiretroviral +Africa+ retention/attrition/adherence/mortality/efficacy/ evaluation - antiretroviral + developing countries + adherence/mortality |
| ISI Web of Science (Science Citation Index and Social Science Citation Index) | - antiretroviral +Africa+ retention/attrition/adherence/mortality/efficacy/ evaluation - antiretroviral + developing countries + adherence/mortality |
| EMBASE | - antiretroviral +Africa+ retention/attrition/adherence/mortality/efficacy/ evaluation - antiretroviral + developing countries + adherence/mortality |
| Cumulative Index to Nursing & Allied Health Literature | - antiretroviral +Africa+retention/attrition/adherence/mortality - antiretroviral + developing countries + adherence/mortality |
| Cochrane Database of Systematic Reviews (up to 2nd quarter 2007) | - antiretroviral +Africa+adherence/mortality |
| IAS abstract archives inception-2006 | - retention - attrition - lost to follow up |
| CROI abstract archives inception-2007 | - retention - attrition - lost to follow up |
| Implementers’ Meeting abstract archives 2006-2007 | - retention - attrition - lost to follow up |
| South Africa AIDS Conference abstract archives 2005-2007 | - retention - attrition - lost to follow up |

Note: for all abstracts that meet the inclusion criteria, search Medline by author to determine if results have been published.

*Bibliographies of published reviews of treatment efficacy and adherence*

- Akileswaran C, Lurie MN, Flanigan TP, Mayer KH. Lessons learned from use of highly active antiretroviral therapy in Africa. *Clin.Infect.Dis*. 2005;**41(3)**:376-85.
- Braitstein P, Brinkhof MW, Dabis F, Schechter M, Boulle A, Miotti P et al. Mortality of HIV-1-infected patients in the first year of antiretroviral therapy: comparison between low-income and high-income countries. *Lancet* 2006;**367(9513)**:817-24.
- Gill CJ, Hamer D, Simon JL, Thea DM, Sabin L. No room for complacency about adherence to antiretroviral therapy in sub-Saharan Africa. *AIDS* 2005;**19**:1243-9.
- Ivers LC, Kendrick D, Doucette K. Efficacy of antiretroviral therapy programs in resource-poor settings: a meta-analysis of the published literature. *Clin.Infect.Dis*. 2005;**41(2)**:217-24.
- Mills EJ, Nachega JB, Buchan I, Orbinski J, Attaran A, Singh S et al. Adherence to antiretroviral therapy in sub-Saharan Africa and North America: a meta-analysis. *JAMA* 2006;**296(6)**:679-90.

Study selection criteria

Inclusion criteria

- Geographic range: sub-Saharan Africa
- Topic: treatment of HIV/AIDS with highly active antiretroviral therapy (ART) (3 drug combination, HIV-1 only)
- Design: observational cohort, intention to treat (must report on outcomes for all patients who discontinued treatment for any reason)
- Setting: standard service delivery in any sector (public, nongovernmental, private)
- Population: adult patients who initiated first-line ART
- Follow up period: minimum median follow up of 6 full months (26 weeks); median follow up period must be clearly specified or able to be calculated from information provided.
- Minimum information included: must report all-cause attrition rate at 6, 12, or 24 months or at the end of the median follow up period or provide information needed to calculate one of these rates.

Exclusion criteria:

- Geographic range: outside sub-Saharan Africa
- Topic: anything other than treatment of HIV-1 with 3-drug ART
- Design: does not observe a cohort or reports only on treatment results or on treatment plus mortality results
- Setting: clinical trial, including phase 3 trials, or other non-standard setting
- Population: pediatric patients or subsets of general treatment-eligible population (e.g. patients known to have drug resistance or specific CD4 counts)
- Follow up period: median of less than 6 full months (26 weeks), including those reporting to 24 weeks, or not specified and not able to be calculated from information provided
- Minimum information included: does not report all-cause attrition rate at 6, 12, or 24 months or at the end of the median follow up period (and none of these rates can be calculated from information reported).

Study quality assessment

Because the review aims to assess public health practice, sources will not be excluded on the basis of quality provided that they meet all the inclusion criteria and none of the exclusion criteria for the review.

First author will do initial screen of sources and assess for inclusion. Co-authors will screen those found to meet the inclusion criteria. Disagreements will be resolved by majority vote (2 of 3 authors).

If more than one source reports on the same cohort of patients, include the one that contains the most complete data about patient retention and attrition over the longest period of follow up.

Data extraction strategy

The following fields will be extracted for each source reviewed:

- Source reference
- Source type (journal article, abstract)
- Country
- Location/site (within country)
- Sector
- Facility level (hospital, clinic, other)
- # facilities included
- Provider
- Treatment funding source
- Patient payment required? (none/partial/full)
- Definition of loss to follow up (LTFU) used
- Dates of observation
- Year enrolment started
- # subjects starting ART
- % started on triple therapy (HAART)
- Age at enrolment (median)
- % female
- Starting CD4 (median)
- Follow up period (median # months)
- Status at end of follow up period:
- Alive and on ART at site (# subjects)
- Transfered (# subjects)
- Total alive and on ART at end of period (# subjects)
- Deaths (# subjects)
- LTFU (# subjects)
- Stopped treatment (# subjects)
- All-cause attrition (# subjects)
- % retained at 6 mos
- % retained at 12 mos
- % retained at 24 mos
- % retained at 36 mos
- % retained at 48 mos.

Plan for synthesis of extracted data

- Report attrition at 6, 12, and 24 month intervals on treatment (plus 36 and 48 months if data allow).
- As needed, estimate attrition at 6, 12, or 24 months based on data provided.
- Estimate average retention at each interval.
- Plot Kaplan-Meier survival curves.
- Use regression to relate cohort characteristics to attrition rates.
- Sensitivity analysis to predict future retention in best- and worst-case scenarios.
